# BMI changes and the risk of lung cancer in male never‐smokers: A prospective cohort study

**DOI:** 10.1002/cam4.4546

**Published:** 2022-01-31

**Authors:** Zheng Wu, Shuanghua Xie, Fei Wang, Shuohua Chen, Kai Su, Fang Li, Hong Cui, Wei Cao, Yiwen Yu, Chao Qin, Yadi Zheng, Xuesi Dong, Zhuoyu Yang, Zilin Luo, Liang Zhao, Yongjie Xu, Hongda Chen, Jiang Li, Gang Wang, Shouling Wu, Min Dai, Ni Li, Jie He

**Affiliations:** ^1^ Office of Cancer Screening, National Cancer Center/ National Clinical Research Center for Cancer/Cancer Hospital Chinese Academy of Medical Sciences and Peking Union Medical College Beijing China; ^2^ Department of Central Laboratory Beijing Obstetrics and Gynecology Hospital, Capital Medical University. Beijing Maternal and Child Health Care Hospital Beijing China; ^3^ Department of Oncology Kailuan General Hospital Tangshan China; ^4^ Department of Thoracic Surgery, National Cancer Center/National Clinical Research Center for Cancer/Cancer Hospital Chinese Academy of Medical Sciences and Peking Union Medical College Beijing China; ^5^ Chinese Academy of Medical Sciences Key Laboratory for National Cancer Big Data Analysis and Implement Chinese Academy of Medical Sciences and Peking Union Medical College Beijing China

**Keywords:** body mass index changes, cancer risk factors, Chinese males, cohort study, epidemiology and prevention, lung cancer, lung cancer risk, never‐smokers, risk assessment

## Abstract

**Background:**

To investigate the association between the risk of lung cancer and short‐term body mass index (BMI) changes in male never‐smokers of a large population‐based prospective study.

**Methods:**

A total of 37,085 male never‐smokers from Kailuan cohort with at least ≥2 BMI measurements were recruited in the present study. The BMI change in the follow‐up was calculated as the annual percent change between BMI at last examination and that at baseline, and categorized into five groups: stable (−0.1 to <0.1 kg/m^2^/year), minor loss (−1.0 to <0.1 kg/m^2^/year) or gain (0.1 to <1.0 kg/m^2^/year), and major loss (<−1.0 kg/m^2^/year) or gain (≥1.0 kg/m^2^/year). The hazards ratios (HRs) and its 95% confidence intervals (CI) were estimated using Cox regression models.

**Results:**

During a median follow‐up of 5.16 years, 224 lung cancer cases were identified. We found a U‐shaped association between BMI changes and lung cancer risk. Compared to men with stable BMI, those with major loss had a nearly twofold higher risk of lung cancer (HR = 1.97, 95% CI: 1.12–3.45), as well as those with major gain had more than twofold higher risk of lung cancer (HR = 2.15, 95% CI: 1.15–4.02). The associations existed when the analysis was stratified by BMI, waist circumference and blood lipids, and lipoproteins concentration at baseline examination.

**Conclusions:**

The dramatic changes in BMI, both gain and loss, might increase lung cancer risk. The control of body weight would be a potential way for lung cancer prevention especially for the nonsmokers.

## Graphical abstract

We investigated the association between the risk of lung cancer and short‐term body mass index (BMI) changes. A total of 37,085 male never‐smokers from a perspective cohort were included; the hazards ratios and 95% confidence intervals were estimated using Cox regression models, and the association between dramatic changes in BMI and the increasing lung cancer risk was found.

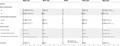

## 1 BACKGROUND

Lung cancer remains the leading cause of mortality worldwide.^1^ China, home to the world's largest population, accounts for 37% of annual global lung cancer incident cases.^2^ Epidemiological studies have recognized smoking as the major cause of lung cancer. Additionally, exposure to air pollution, radon gas, and asbestos or other pollutants can also increase the risk of lung cancer.^3^, ^4^, ^5^, ^6^ However, the established risk factors could not explain all the lung cancer occurrence.

Since 1980s, the body weight and fatness were found to be correlated with the risk of lung cancer.^7^ Known as an indicator of body fatness, body mass index (BMI) has been used widely for assessing the relationship between body weight and fatness and the cancer risk.^8^ So far, a number of epidemiological studies have shown that BMI was negatively associated with lung cancer,^9^, ^10^, ^11^, ^12^, ^13^ which indicates that higher BMI may decrease the lung cancer risk, or lower BMI may increase the lung cancer risk.^9^, ^12^

The measurement of body weight at a single time point may not be sufficient for assessing the relationship between body weight and cancer risk due to the fact that body weight changes over time. Involving BMI changes during a specific time period would be informative.^14^ Eleven studies, including seven cohort studies^7^, ^15^, ^16^, ^17^, ^18^, ^19^, ^20^ and four case–control studies^14^, ^21^, ^22^, ^23^ have evaluated the correlation between BMI changes and lung cancer risk until today, but the findings were also not consistent. Eight studies of 11^7^, ^16^, ^17^, ^18^ showed an inverse association of long‐term (over several decades) BMI change with lung cancer incidence risk, while one study^20^ reported a positive association of short‐term (6 years) BMI change. Additionally, the other two studies^15^, ^19^ did not find BMI change to be associated with lung cancer risk.

Thus far, a paucity of data, especially data from prospective studies, persists in the literature regarding BMI change and the lung cancer risk among nonsmokers because of the limited lung cancer cases.^14^, ^17^ Additionally, the time period of weight change assessment was several decades ranging from the age of 18–20 years to later adulthood in most studies (9/11), while only few studies (2/11) focused on a shorter period (i.e., one decade or less), and the results were also inconsistent. Compared to weight change over long periods, changes in weight during short intervals may be more meaningful because people in their middle age or older might be prone to change their lifestyle behaviors to prevent noncommunicable chronic diseases (NCDs) including cancer.^19^

In terms of these knowledge gaps, we aimed to investigate the relationship between short‐term BMI changes––longitudinal measured BMI change over 2–5 years––and lung cancer in male never‐smokers based on a Chinese cohort in this study. This prospective study would provide evidence on the effect of short‐term BMI change on the lung cancer risk.

## 2 METHODS

### 2.1 Study design and population

The Kailuan study was an ongoing prospective dynamic cohort study conducted in Tangshan, China, which aimed to investigate the risk factors for NCDs including cancer. The rationale and methodology were introduced previously.^24^, ^25^, ^26^ Briefly, currently working and retired employees who were older than 18 years from the Kailuan Group were enrolled to undergo questionnaire interviews and clinical examinations biennially since May 2006. Individuals missing the previous health examination could undertake the next round of examination.

The present study was restricted to males with at least two BMI measurements made during health examinations performed in 2006–2007, 2008–2009, or 2010–2011, with changes in BMI assessed from the first time point to the second time point (baseline of this study) (*n* = 76,518). Of the 76,518 males, we excluded those who missed the date of examination (*n* = 3808), had extreme BMI values at any health examinations (BMI < 15 or >45 kg/m^2^) (*n* = 63), diagnosed with cancer before enrollment, or during the period when BMI change was assessed (*n* = 590), and ever smokers (*n* = 34,972). Finally, a total of 37,085 never‐smokers were included in this study. In addition, as a sensitivity analysis, underweight individuals (BMI < 18.5 kg/m^2^) at the first or last examination were further excluded to minimize the influence of preexisting disease (*n* = 741). Participants developing lung cancer within 1 year after the BMI change assessment were also excluded to eliminate the reverse association (*n* = 41).

All individual participants gave written informed consent according to the guidelines of the Helsinki declaration, and the study protocol was approved by the Medical Ethics Committee of Kailuan General Hospital.

### 2.2 Assessment of BMI and BMI changes

Measurements of height and body weight were conducted by trained medical assistants according to standard operating procedures. In brief, portable stadiometers were used to measure height, and calibrated platform scales were used for measuring body weight. Both the weight and height of all the participants were measured twice without heavy clothing or any other accessories, for example, shoes and hats. BMI was calculated as weight (kg)/height^2^ (m^2^).

Based on the study performed by Rapp et al,^19^ BMI changes were calculated as the annual change in BMI from the first to the last examination, which was calculated by the formula as follows: [(BMI at the last examination − BMI at the first examination)/(years between the first and the last examination)]. In our analysis, we also found that the risk of lung cancer within −0.1 to <0.1 was relatively stable, while the risk of lung cancer <−0.1 or ≥ 0.1 was increased (Figure S2), similar with former research.^19^ Thus the changes of BMI were categorized into groups of stable (≥−0.1 and <0.1 kg/m^2^/year), minor loss (≥−1.0 and <0.1 kg/m^2^/year) or gain (≥ 0.1 and <1.0 kg/m^2^/year), and major loss (<−1.0 kg/m^2^/year) or gain (≥1.0 kg/m^2^/year), and stable group was served as the reference category.

### 2.3 Assessment of covariates

The definitions and classifications of covariates including income levels, education levels, alcohol consumption and coal mine dust exposure, and details of measurements of blood lipids and lipoproteins (i.e., total cholesterol [TC], low‐density lipoprotein cholesterol [LDL‐C], and triglycerides [TG]) were introduced in our previous study.^26^ According to the Chinese guideline for the management of dyslipidemia in adults,^27^ TC was defined as abnormal if the concentration was greater than 5.2 mg/dl, TG was defined as abnormal if the concentration was >1.7 mg/dl, and LDL‐C was defined as abnormal if the concentration was >3.4 mg/dl. The total blood lipids were classified as abnormal if any of the three types of blood lipids and lipoproteins indicated abnormality.

### 2.4 Outcome ascertainment

Participants were followed beginning at the baseline (after BMI change was assessed) and ending at the first diagnosis of cancer, death, or December 31, 2015, whichever event occurred first. New lung cancer cases were identified through questionnaire interviews and clinical examinations biennially. Additionally, incident lung cancer cases could be tracked yearly through search of medical records linked with the Tangshan medical insurance system and the Kailuan social security system. Furthermore, we also checked discharge summaries annually from the 11 affiliated hospitals to obtain the outcome information.

The diagnosis of lung cancer was confirmed by clinical experts' review of medical records. Information on imaging diagnosis (e.g., computerized tomography) and pathological diagnosis was collected to assess lung cancer incidence. All lung cancer events were coded as C34 in accordance with the International Classification of Diseases, Tenth Revision (ICD‐10).

### 2.5 Statistical analyses

Categorical variables were described and compared by percentages and chi‐square test. Continuous variables were described and compared by the mean (standard deviation) and ANOVA. Cox proportional hazards regression models were used to estimate the association of BMI change with lung cancer incidence risk. Person‐years at risk was computed from the date of baseline until the date of ending follow‐up in this study. Multivariable Cox proportional hazards regression models were conducted adjusting for age (continuous), income levels (<500, 500–1000, and >1000 Chinese Yuan [CNY]/month), education levels (illiterate or primary school, junior high school, senior high school, and college and above), alcohol drinking (ever‐ and never‐ drinker), coal dust exposure (nonexposure or exposure), degree of coal dust exposure (light, moderate, or heavy), dietary fat intake (never, <3 times/week, or ≥3 times/week), physical activity (never, <4 times/week, or ≥4 times/week), waist circumference (continuous), and BMI at the baseline examination (continuous). As TC, LDL‐C, and TG were found to be correlated with increased lung cancer risk particularly in male never‐smokers,^26^ we further adjusted for the serum concentrations of these three types of blood lipids and lipoproteins. Tests of linear trends for BMI changes were performed by entering the categories as a continuous variable in the regression model, and restricted spline curves were used to display the results directly. We also tested the linear trend of BMI (continuous), as a supplementary analysis.

Subgroup analyses were performed by waist circumference (<85 cm and ≥85 cm) and blood lipids and lipoproteins status (normal vs. abnormal).

Sensitivity analyses were conducted to examine the consistency of our findings. First, lung cancer cases diagnosed within the first year of follow‐up were excluded from the analyses to evaluate the possibility of reverse causation. Second, given that preexisting diseases may result in weight loss and thus the association of BMI loss with lung cancer risk may be overestimated, we repeated the main models after excluding subjects with BMI < 18.5 kg/m^2^ at the first or last examination. Third, participants with coal dust exposure were not included in the analyses to exclude the impact of occupational exposure on the risk of lung cancer.

The data management and all analyses were conducted using SAS statistical software, version 9.4 (SAS Institute Inc.). All statistical tests were two‐sided, and *p* < 0.05 was regarded as statistically significant.

## 3 RESULTS

### 3.1 Baseline participant characteristics

**FIGURE 1:**
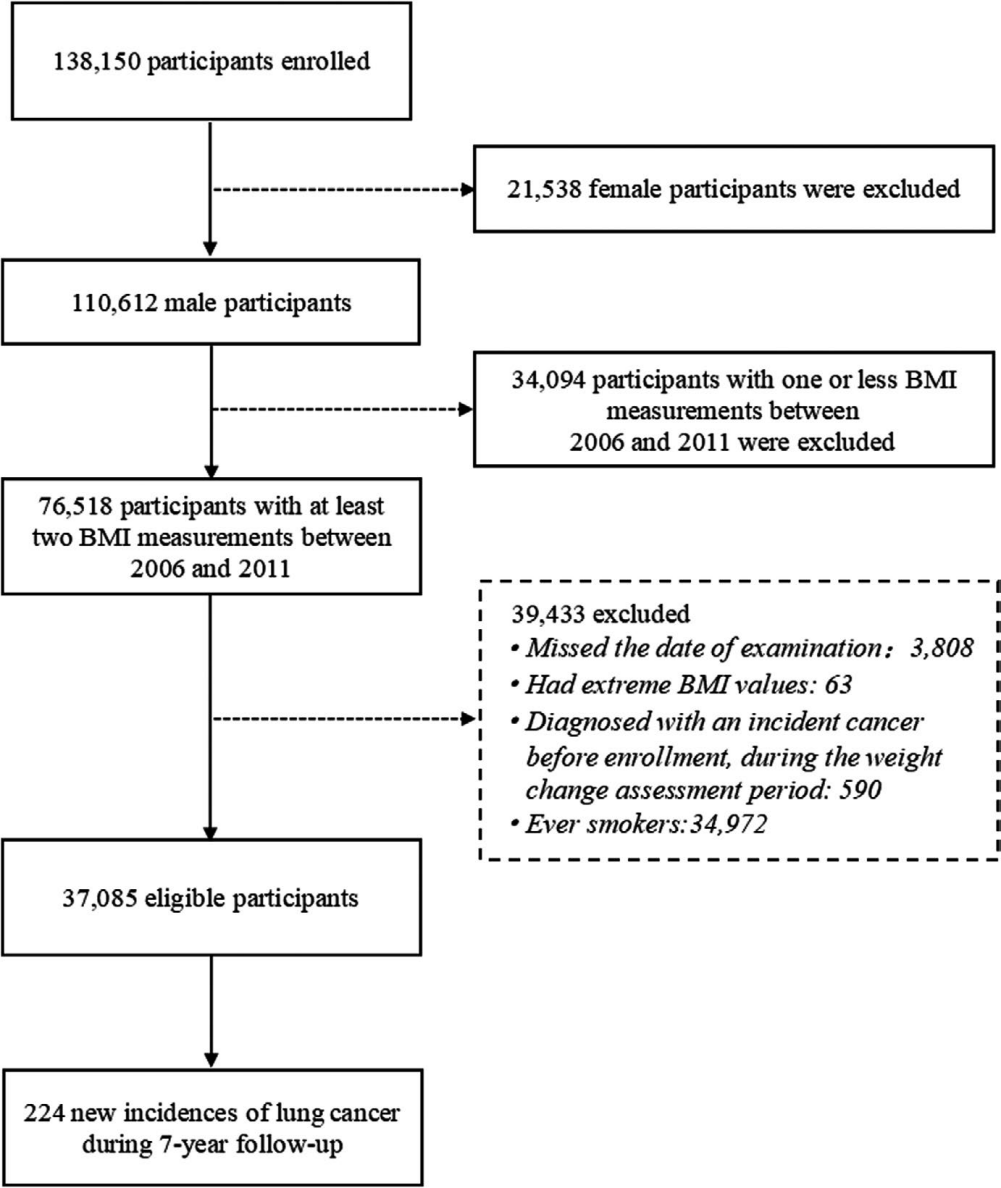
Flowchart of the selection of participants, Kailuan cohort, 2006–2015

A total of 37,085 participants were included in the present study with the mean age of 51.27 years (Figure 1). The mean BMI and waist circumference were 25.31 kg/m^2^ and 88.25 cm, respectively, and mean concentrations of TC, LDL‐C, and TG were 4.89, 2.35, and 1.71 mg/dl, respectively. The rates of alcohol drinking and coal dust exposure were 9.83% and 60.37%, respectively (Table 1).

**TABLE 1:** Baseline characteristics of male never‐smokers by lung cancer, Kailuan cohort, 2006–2015

Characteristics	Total cohort	Lung cancer		*p* ^a^
		Yes	No	
No.	37,085	224	36,861	
Age (years), mean (*SD*)	51.27 ± 12.76	58.60 ± 9.63	51.23 ± 12.77	<0.001
BMI (kg/m^2^), mean (*SD*)	25.31 ± 3.34	24.81 ± 3.13	25.31 ± 3.34	0.032
WC (cm), mean (*SD*)	88.25 ± 9.51	89.51 ± 10.03	88.24 ± 9.51	0.101
TC (mg/dl), mean (*SD*)	4.89 ± 1.15	4.97 ± 1.06	4.89 ± 1.15	0.156
TG (mg/dl), mean (*SD*)	1.71 ± 1.46	1.67 ± 1.41	1.71 ± 1.46	0.305
LDL‐C (mg/dl), mean (*SD*)	2.35 ± 0.98	2.31 ± 1.03	2.35 ± 0.97	0.516
Education level, No. (%)				0.002
Illiterate or primary school	3306 (8.93)	22 (9.87)	3,284 (8.92)	
Junior high school	27,547 (74.38)	185 (82.96)	27,362 (74.33)	
Senior high school	3824 (10.33)	12 (5.38)	3812 (10.36)	
College and above	2357 (6.36)	4 (1.79)	2353 (6.39)	
Income/month (CNY), No. (%)				0.004
<500	7251 (19.65)	35 (15.91)	7216 (19.68)	
500–1000	24,648 (66.81)	169 (76.82)	24,479 (66.75)	
≥1000	4994 (13.54)	16 (7.27)	4978 (13.57)	
Alcohol consumption status, No. (%)				0.369
Never‐drinker	33,441 (90.17)	198 (88.39)	33,243 (90.18)	
Ever drinker	3644 (9.83)	26 (11.61)	3618 (9.82)	
Coal dust exposure status, No. (%)				0.001
Nonexposed	14,674 (39.63)	113 (50.67)	14,561 (39.56)	
Exposed	22,356 (60.37)	110 (49.33)	22,246 (60.44)	
Degree of coal dust exposure, No. (%)				0.964
Light	11,744 (52.53)	59 (53.64)	11,685 (52.53)	
Moderate	4476 (20.02)	22 (20.00)	4454 (20.02)	
Heavy	6136 (27.45)	29 (26.36)	6107 (27.45)	
Physical activity, No. (%)				0.183
Never	3284 (8.87)	12 (5.38)	3272 (8.89)	
<4 times/week	29,146 (78.69)	183 (82.06)	28,963 (78.67)	
≥4 times/week	4607 (12.44)	28 (12.56)	4579 (12.44)	
Dietary fat intake, No. (%)				0.340
Never	2773 (7.50)	12 (5.38)	2761 (7.51)	
<3 times/week	32,122 (86.85)	201 (90.13)	31,921 (86.83)	
≥3 times/week	2090 (5.65)	10 (4.48)	2080 (5.66)	
Annual BMI change (kg/m^2^/year), No. (%)^b^				0.008
Major loss	2481 (6.69)	26 (11.61)	2455 (6.66)	
Minor loss	13,007 (35.07)	76 (33.93)	12,931 (35.08)	
Stable	6142 (16.56)	25 (11.16)	6117 (16.59)	
Minor gain	13,253 (35.74)	79 (35.27)	13,174 (35.74)	
Major gain	2202 (5.94)	18 (8.04)	2184 (5.92)	

Abbreviations: BMI, body mass index; CNY, Chinese yuan; LDL‐C, low‐density lipoprotein cholesterol; TC, total cholesterol; TG, triglycerides; WC, waist circumference.

^a^ *p* values from ANOVA (continuous variables) or chi‐square test (categorical variables).

^b^ Major loss (<−1.0 kg/m^2^/year), minor loss (≥−1.0 kg/m^2^/year and <−0.1 kg/m^2^/year), sable (≥−0.1 kg/m^2^/year and <0.1 kg/m^2^/year), minor gain (≥0.1 kg/m^2^/year and <1.0 kg/m^2^/year), major gain (≥1.0 kg/m^2^/year).

By December 2015, 224 incident lung cancer cases were verified during a median follow‐up of 5.16 years. Compared to participants without lung cancer, those with lung cancer were prone to be older, have a slightly lower BMI, lower education levels, lower income levels, and were less likely to expose to coal dust (all *p* values < 0.05) at baseline examination. The waist circumference, blood lipids and lipoproteins concentrations, and rates of alcohol drinking, physical activity, and dietary fat intake were comparable between these two groups (all *p* values > 0.05). (Table 1).

Annual BMI change during 2006–2010 were stable in 16.56% (*n* = 6142) of males. Minor and major BMI loss occurred in 35.07% (*n* = 13,007) and 6.69% (*n* = 2481) of males, respectively, and minor and major BMI gain occurred in 35.74% (*n* = 13,253) and 5.94% (*n* = 2202) of males, respectively. Baseline characteristics of the study population by the categories of annual BMI change were listed in Table 2. Men who had a major BMI gain were more likely to have normal BMI (<25 kg/m^2^), higher waist circumference (≥85 cm), normal TC concentration (<5.2 mg/dl), and normal TG concentration (<1.7 mg/dl) at baseline, while those had a major BMI loss were more likely have higher BMI (≥25 kg/m^2^), higher waist circumference (≥85 cm), and higher TG concentration (≥1.7 mg/dl) (all *p* values < 0.05) (Table 2).

**TABLE 2:** Baseline characteristics of male never‐smokers by categories of annual BMI changes, Kailuan cohort, 2006–2015

Characteristics	Annual BMI changes (kg/m^2^/year)^a^					*p* ^b^
	Major loss	Minor loss	Stable	Minor gain	Major gain	
No.	2481	13,007	6142	13,253	2202	
Age (years), mean (*SD*)	52.28 ± 12.98	51.47 ± 12.25	51.10 ± 12.37	50.93 ± 13.07	51.57 ± 14.5	<0.001
BMI (kg/m^2^), No. (%)						<0.001
<18.5	3 (0.12)	50 (0.38)	62 (1.01)	253 (1.91)	109 (4.95)	
≥18.5 and <25.0	436 (17.57)	4908 (37.73)	2975 (48.44)	7525 (56.78)	1437 (65.26)	
≥25.0 and <30.0	1353 (54.53)	6752 (51.91)	2764 (45.00)	4825 (36.41)	572 (25.98)	
≥30.0	689 (27.77)	1297 (9.97)	341 (5.55)	650 (4.90)	84 (3.81)	
WC (cm), No. (%)						<0.001
<85	639 (25.76)	4114 (31.63)	2150 (35.00)	4953 (37.37)	855 (38.83)	
≥85	1842 (74.24)	8893 (68.37)	3992 (65.00)	8300 (62.63)	1347 (61.17)	
TC (mg/dl), No. (%)						<0.001
<5.2	1643 (66.22)	8402 (64.60)	3969 (64.62)	8656 (65.31)	1542 (70.03)	
≥5.2	838 (33.78)	4605 (35.40)	2173 (35.38)	4597 (34.69)	660 (29.97)	
TG (mg/dl), No. (%)						<0.001
<1.7	1590 (64.09)	8539 (65.65)	4210 (68.54)	9312 (70.26)	1567 (71.16)	
≥1.7	891 (35.91)	4468 (34.35)	1932 (31.46)	3941 (29.74)	635 (28.84)	
LDL‐C (mg/dl), No. (%)						0.041
<3.4	2217 (89.36)	11,663 (89.67)	5574 (90.75)	11,990 (90.47)	1972 (89.55)	
≥3.4	264 (10.64)	1344 (10.33)	568 (9.25)	1263 (9.53)	230 (10.45)	
Education level, No. (%)						<0.001
Illiterate or primary school	240 (9.69)	1151 (8.86)	476 (7.76)	1232 (9.31)	207 (9.43)	
Junior high school	1910 (77.08)	9760 (75.11)	4611 (75.16)	9658 (73.00)	1608 (73.22)	
Senior high school	222 (8.96)	1334 (10.27)	623 (10.15)	1419 (10.72)	226 (10.29)	
College and above	106 (4.28)	749 (5.76)	425 (6.93)	922 (6.97)	155 (7.06)	
Income/month (CNY), No. (%)						<0.001
<500	359 (14.59)	2745 (21.21)	1260 (20.60)	2636 (19.98)	251 (11.51)	
500–1000	1687 (68.58)	8638 (66.75)	4123 (67.40)	8760 (66.39)	1440 (66.02)	
≥1000	414 (16.83)	1558 (12.04)	734 (12.00)	1798 (13.63)	490 (22.47)	
Alcohol consumption status, No. (%)						<0.001
Never‐ drinker	2305 (92.91)	11,663 (89.67)	5545 (90.28)	11,915 (89.90)	2013 (91.42)	
Ever drinker	176 (7.09)	1344 (10.33)	597 (9.72)	1338 (10.10)	189 (8.58)	
Coal dust exposure status, No. (%)						<0.001
Nonexposed	1090 (43.99)	4808 (37.01)	2335 (38.06)	5432 (41.06)	1009 (45.95)	
Exposure	1388 (56.01)	8184 (62.99)	3800 (61.94)	7797 (58.94)	1187 (54.05)	
Degree of coal dust exposure, No. (%)						<0.001
Light	799 (57.56)	4295 (52.48)	1957 (51.50)	4001 (51.31)	692 (58.30)	
Moderate	229 (16.50)	1672 (20.43)	794 (20.89)	1586 (20.34)	195 (16.43)	
Heavy	360 (25.94)	2217 (27.09)	1049 (27.61)	2210 (28.34)	300 (25.27)	
Physical activity, No. (%)						0.007
Never	221 (8.92)	1088 (8.37)	506 (8.25)	1257 (9.50)	212 (9.65)	
<4 times/week	1964 (79.26)	10,273 (79.06)	4841 (78.91)	10,321 (78.00)	1747 (79.48)	
≥4 times/week	293 (11.82)	1633 (12.57)	788 (12.84)	1654 (12.50)	239 (10.87)	
Dietary fat intake, No. (%)						<0.001
Never	189 (7.64)	923 (7.12)	440 (7.18)	1037 (7.84)	184 (8.38)	
<3 times/week	2173 (87.87)	11,360 (87.57)	5329 (87.00)	11,351 (85.87)	1909 (86.93)	
≥3 times/week	111 (4.49)	689 (5.31)	356 (5.81)	831 (6.29)	103 (4.69)	

Abbreviations: BMI, body mass index; CNY, Chinese yuan; LDL‐C, low‐density lipoprotein cholesterol; TC, total cholesterol; TG, triglycerides; WC, waist circumference.

^a^ Major loss (<−1.0 kg/m^2^/year), minor loss (≥−1.0 kg/m^2^/year and <−0.1 kg/m^2^/year), stable (≥−0.1 kg/m^2^/year and <0.1 kg/m^2^/year), minor gain (≥0.1 kg/m^2^/year and <1.0 kg/m^2^/year), major gain (≥1.0 kg/m^2^/year).

^b^ *p* values from chi‐square tests.

### 3.2 Association between changes in BMI and lung cancer risk

**FIGURE 2:**
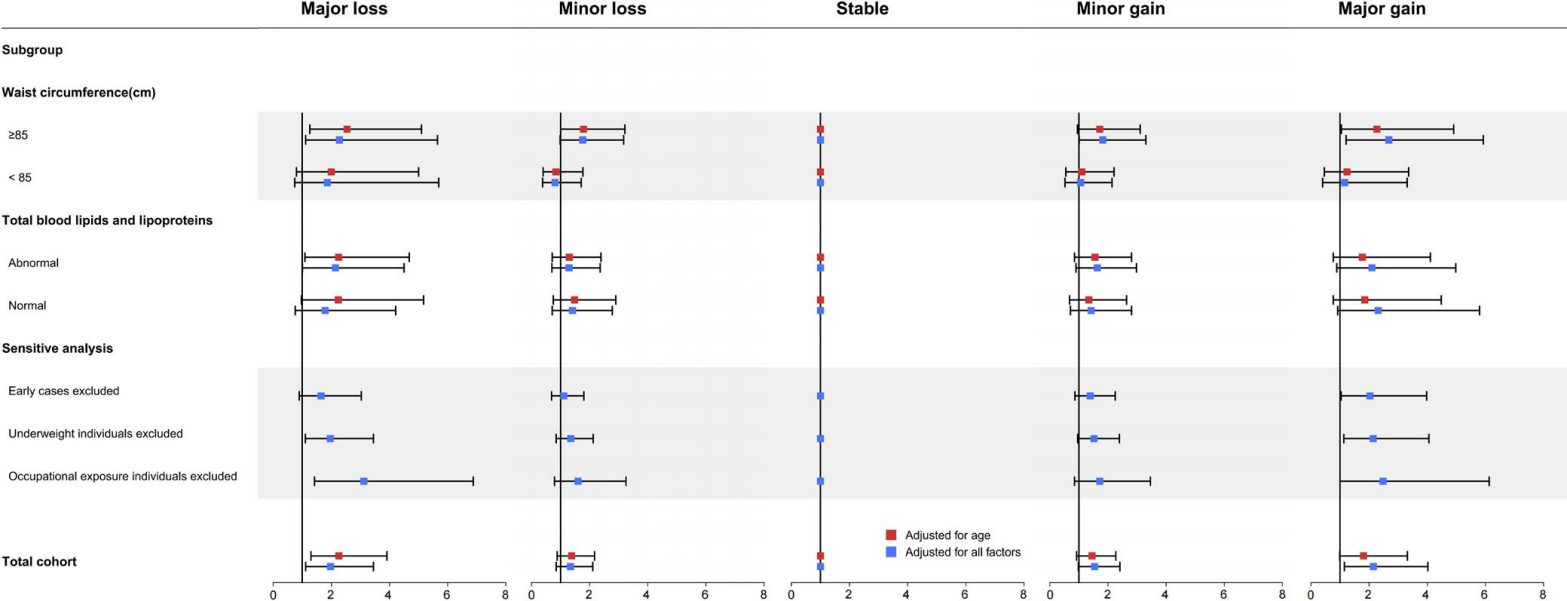
Hazard ratio and 95% confidence interval of the association between body mass index changes and lung cancer risk in the whole cohorts and subgroups, and sensitive analysis, Kailuan cohort, 2006–2015

Compared with males with stable BMI (average annual change between −0.1 and <0.1 kg/m^2^/year), major BMI changes, both major loss and major gain, were observed to be significantly associated with lung cancer risk with a positive dose–response relation. Relative to males with stable BMI, those with major BMI gain (≥1.0 kg/m^2^/year) had an increased lung cancer risk (hazards ratio [HR] = 1.81, 95% CI: 0.99–3.32) when adjusted for age. The association was strengthened when additionally adjusted for income levels, education levels, alcohol drinking status, coal dust exposure status, degree of coal dust exposure, physical activity, dietary fat intake, TC, TG, LDL‐C, waist circumference, and BMI at baseline (HR = 2.15, 95% CI: 1.15–4.02). Males who experienced major BMI loss (<−1.0 kg/m^2^/year) were also more likely to develop lung cancer (HR = 2.26, 95% CI: 1.30–3.91) when adjusted for age and the association was were attenuated yet still statistically significant in multivariable‐adjusted models (HR = 1.97, 95% CI: 1.12–3.45) (Figure 2).

Subgroup analyses showed that major BMI change, both gain and loss increased the lung cancer incidence risk especially in abdominal obesity males (waist circumference ≥85 cm) (for major gain: HR = 2.68, 95% CI: 1.21–5.93; for major loss: HR = 2.28, 95% CI: 1.12–4.65). Same positive associations existed when analysis was stratified by baseline serum total blood lipids and lipoproteins status (normal vs. abnormal). Major BMI change, both gain and loss were correlated with elevated lung cancer risk among males who had normal serum total blood lipids and lipoproteins status (for major gain: HR = 2.31, 95% CI: 0.92–5.80; for major loss: HR = 1.79, 95% CI: 0.76–4.21), as well as among those had abnormal serum blood lipids and lipoproteins status (for major gain: HR = 2.10, 95% CI: 0.89–4.98; for major loss: HR = 2.14, 95% CI: 1.01–4.50) (Figure 2).

In the sensitivity analysis, after excluding lung cancer cases (no. = 41) that occurred within the first 1 year of follow‐up, the results were consistent with the main findings (for major gain: HR = 2.03, 95% CI: 1.04–3.98; for major loss: HR = 1.65, 95% CI: 0.90–3.03). The analysis which excluded subjects who were underweight (BMI <18.5 kg/m^2^) at the first or last examination (*n* = 741, case no. = 4) did not alter the main findings (for major gain: HR = 2.14, 95% CI: 1.13–4.06; for major loss: HR = 1.96, 95% CI: 1.11–3.45). When excluding individuals with coal dust exposure (*n* = 22,356, case no. = 110), compared to individuals with stable BMI, an elevated lung cancer risk was found among males with major BMI gain and loss (for major gain: HR = 2.48, 95% CI: 1.00–6.13; for major loss: HR = 3.12, 95% CI: 1.42–6.88) (Figure 2).

**FIGURE 3:**
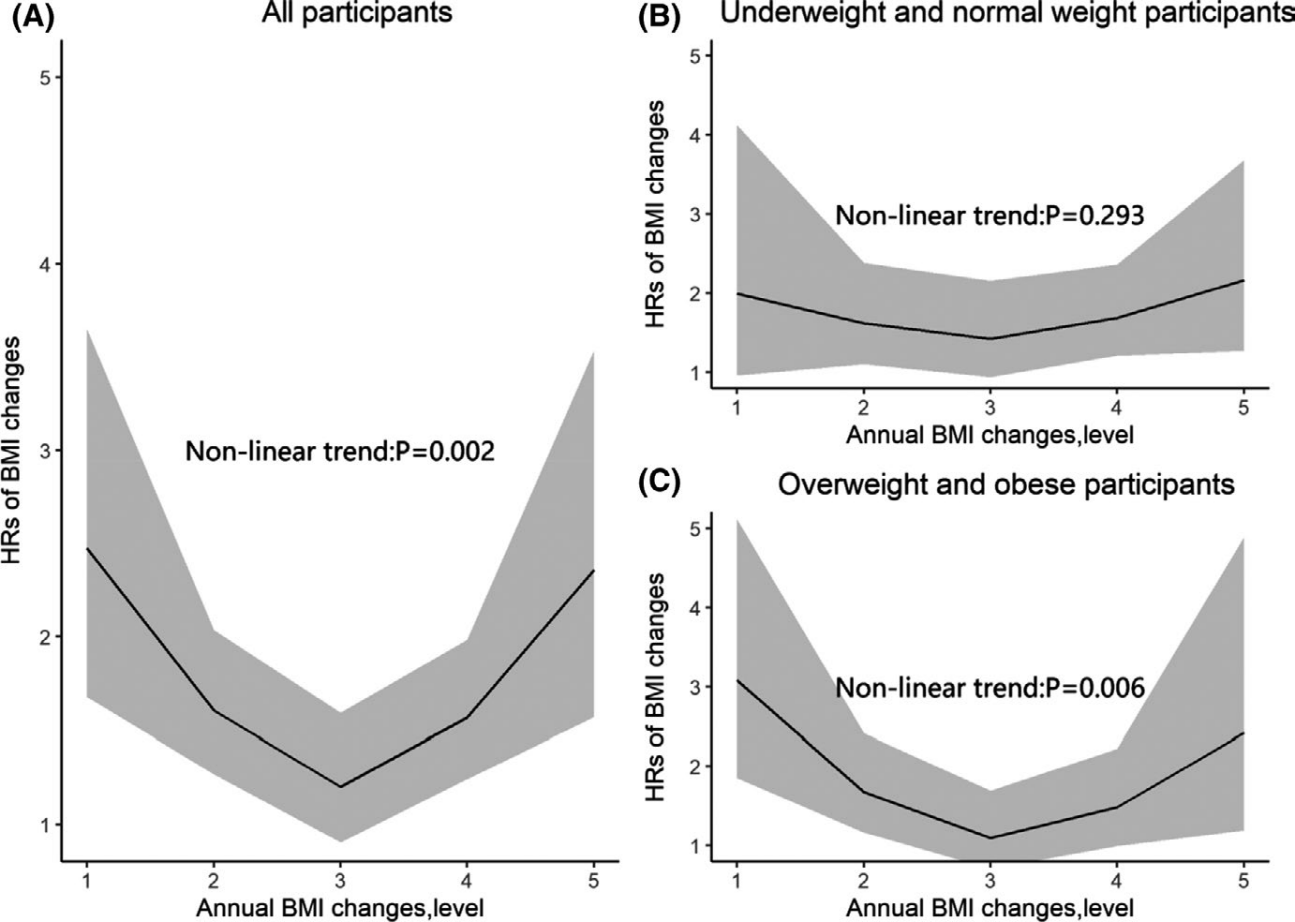
Restricted spline curves for the associations between annual change in body mass index (BMI; entering the categories as a continuous variable) and lung cancer incidence among (A) all participants, (B)under weight and normal, and (C) overweight and obesity participants

After adjusting all selected covariates, the nonlinear relationship between BMI change and the risk of lung cancer were statistically significant among the whole cohort (*p* for nonlinear trend = 0.002; Figure 3A), and the overweight/obese subgroup (*p* for nonlinear trend = 0.006; Figure 3C); the nonlinear relationship was not significant in the under/normal weight subgroup (*p* for nonlinear trend = 0.293; Figure 3B), and in the analysis treated BMI as a continuous variable (Figure S1).

Results did not change appreciably when cancers diagnosed during the first 3 years of follow‐up (vs. the first year of follow‐up) were excluded (data not shown).

## 4 DISCUSSION

This is the first prospective cohort study trying to investigate the association between short‐term BMI changes––longitudinal measured BMI changes over 2–5 years––and lung cancer risk in Chinese male never‐smokers. In the present study, we found that major short‐term changes in BMI, both loss and gain, were positively associated with lung cancer incidence risk with a positive dose–response relation in adult men who never smoked, compared to men with a stable BMI. Major gain of short‐term BMI increased lung cancer risk significantly, as well as major loss in the follow‐up. The associations were unchanged after excluding subjects developing lung cancer within the first 1 year in the follow‐up, participants with BMI <18.5 kg/m^2^ at baseline examination, and individuals with coal dust exposure.

So far, only a few studies have examined BMI or weight changes regarding lung cancer incidence risk and most of the prior studies focused on the long‐term (>20 years) adult changes since early adulthood (ranging from age 18 to 30). Four prospective^7^, ^16^, ^17^, ^18^ and four case–control studies^14^, ^21^, ^22^, ^23^ have reported that long‐term weight or BMI change since early adulthood was inversely associated with lung cancer risk. Taken together, an increased risk was found with long‐term weight loss, although the definition of weight loss differed across studies.^7^, ^18^, ^21^

However, such positive association in the prior studies were observed mainly in current smokers, but not in nonsmokers. Concerned that smoking may lead to residual confounding, thus could distort the observed association since smoking is the main cause for lung cancer and can affect body weight, we investigated the association of BMI changes with lung cancer risk in never‐smokers, and we also found that a major short‐term loss in BMI was correlated with elevated lung cancer risk among male never‐smokers, which was consistent with prior studies. In addition, three prospective^7^, ^16^, ^17^ and four population‐based case–control studies^14^, ^21^, ^22^, ^23^ found long‐term adult weight gain since early adulthood to be negatively correlated with lung cancer, especially in current smokers, but our study found short‐term BMI gain to be positively associated with lung cancer risk, and the same positive association still existed when analysis was stratified by categories of waist circumference and serum blood lipids and lipoproteins concentration at baseline examination, respectively, which was interesting. Thus far, the association of short‐term BMI gain with lung cancer among nonsmokers warranted further research because of the paucity of available evidence in relation to this topic. One prospective study of Swedish male construction workers reported a positive association of short‐term BMI gain after 6 years of follow‐up with lung cancer risk after adjusting for smoking status (never, former, current),^20^ which was in consistence with our findings.

The mechanisms for the observed positive associations of short‐term BMI changes with lung cancer among male never‐smokers is unclear. BMI has been reported with a linear association with adipokine or leptin, which played a critical role in a number of pathways such as inflammatory response, energy regulation, and tumorigenesis, which may associate with the risk of lung cancer.^28^, ^29^, ^30^, ^31^ Hence, we may hypothesize, in bold, that BMI changes may also associate with the change of adipokine or leptin, and the relationships may translate into effects of BMI changes on lung cancer risk, or some other mechanisms underlying the process of short‐term BMI changes in adulthood may be involved in the genesis of lung cancer. In any case, the etiology of short‐term BMI changes among never‐smokers remains unclear. Our research with respect to short‐term BMI changes and risk of lung cancer in nonsmokers is among the first such studies, which might be worth replicating and possibly suggest some future research directions.

It is possible that the observed association may be due to preclinical weight loss in subjects who develop lung cancer after enrollment in our study. However, as a large prospective cohort study, we prospectively measured weight and height individually by trained staff every 2 years, and associations were unchanged after further excluding lung cases verified within the first year of follow‐up and subjects with BMI < 18.5 kg/m^2^ at the first and last examination. In addition, in a prospective study, Abraham et al^7^ indicated that long‐time adult weight change since 25 years old particularly affected lung cancer risk within 5–9 years after examination, but not within 0–4.9 years. All these work suggested that the association, especially the short‐term BMI loss association with lung cancer, could not be totally explained by preclinical effect of cancer. Other studies have reported similar findings.^18^, ^21^

Our results are worth consideration for several reasons. First, to our knowledge, our study is the first to evaluate the association of short‐term BMI changes over 2–5 years with lung cancer in never‐smokers in an Asian population. The second reason might be that a large number of never‐smokers (*n* = 37,085) were included, and the follow‐up rate was high: 95% of participants could be traced back, because the examination was conducted every 2 years and all expenses were paid by the Kailuan Group. Furthermore, weight and height in our study were measured in a standardized way during every examination, which could eliminate measurement bias and misclassification of BMI change category compared with self‐reported weight and height. Comprehensive confounders, such as dietary fat intake, waist circumference, blood lipids, and lipoproteins, were adjusted in our multivariable models, which was also one strength of the study design. In China, confronting the rising of BMI^32^ and incidence rate of lung cancer, our results might have the potential to clarify their associations, and facilitate clinical decision‐making in more cases.

We acknowledge some limitations of the study. The major limitation was the relatively short follow‐up time (median = 5.16 years), however, the age‐adjusted incidence of the lung cancer in the cohort was in line with the national level according to our previous study.^26^ Additionally, some epidemiological studies have shown that environmental tobacco smoke (EVT) was associated with an elevated risk of lung cancer.^33^ However, information on EVT was not available in our study, which could be a concern when interpreting our results. Some factors were proved closely related to lung cancer in recent years, such as passive smoking^34^; but our study began in 2006, and due to the knowledge limitation at that time, information on these factors were not collected.

## 5 CONCLUSIONS

In conclusion, in this relatively large prospective cohort study among Chinese male never‐smokers, we found evidence that major short‐term BMI changes, both loss or gain, were associated with increased lung cancer risk. Further epidemiological and experimental researches will be helpful to confirm the associations and explore the possible biological basis between short‐term BMI changes and lung cancer risk among never‐smokers.

## CONFLICT OF INTEREST

The authors declare that they have no competing interest.

## AUTHOR CONTRIBUTIONS

Conception and design: NL, MD, JH, and SW. Acquisition of data: ZW, SX, FW, KS, HC, GW, FL, and SC. Analysis of data: ZW, SX, FW, CQ, YZ, ZY, and ZL. Interpretation of data: WC, HC, YY, CQ, XD, LZ, and YX. Writing, review, and/or revision of the manuscript: ZW, SX, FW, NL, and MD. Administrative, technical, or material support: FT, KS, and FL. Study supervision: NL, MD, and JH. All authors approved the final version of the manuscript.

## ETHICS APPROVAL AND CONSENT TO PARTICIPATE

The studies involving human participants were reviewed and approved by the ethic committee of the Kailuan General Hospital. The patients/participants provided their written informed consent to participate in this study.

## CONSENT FOR PUBLICATION

Not applicable.

## Supporting information


Figure S1
Figure S2

## Data Availability

The datasets for this manuscript are not publicly available because all our data are under regulation of both the National Cancer Center of China and Kailuan Group. Requests to access the datasets should be directed to JH, prof.jiehe@gmail.com and SLW, drwusl@163.com.
