# Supplementary material for: BMI changes and the risk of lung cancer in male never‐smokers: A prospective cohort study
Source: Cancer Med. 2022 Jan 31;11(5):1336–46. doi: 10.1002/cam4.4546 (PMC8894701; doi:10.1002/cam4.4546)
Supplement: Supplementary file 1 — Figure S1 Figure S2 [file CAM4-11-1336-s001.docx]

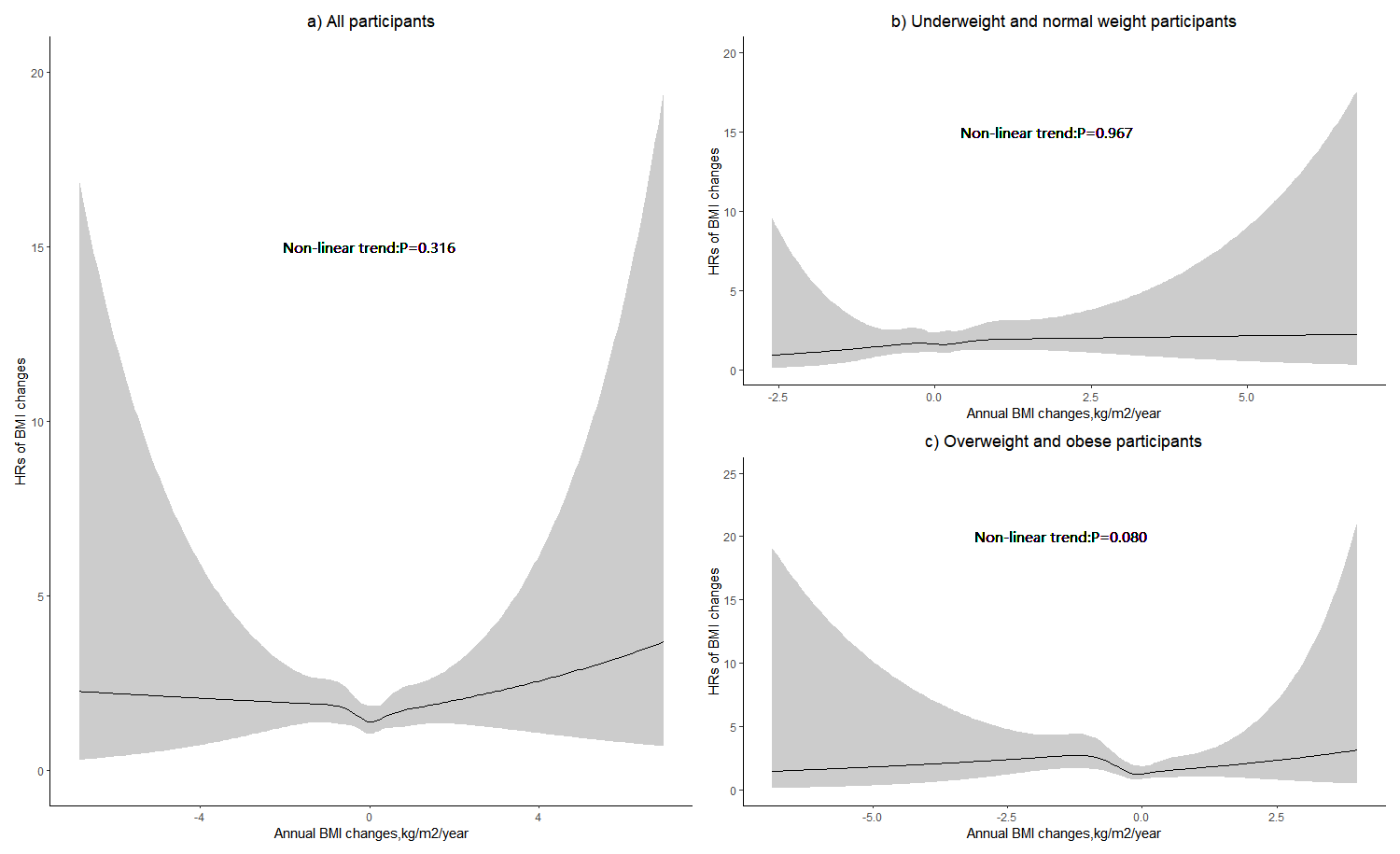


**Supplement Figure 1.** Restricted spline curves for the associations between annual change in BMI(continuous) and lung cancer incidence among a)all participants, b)under weight and normal, and c)overweight and obesity participants.


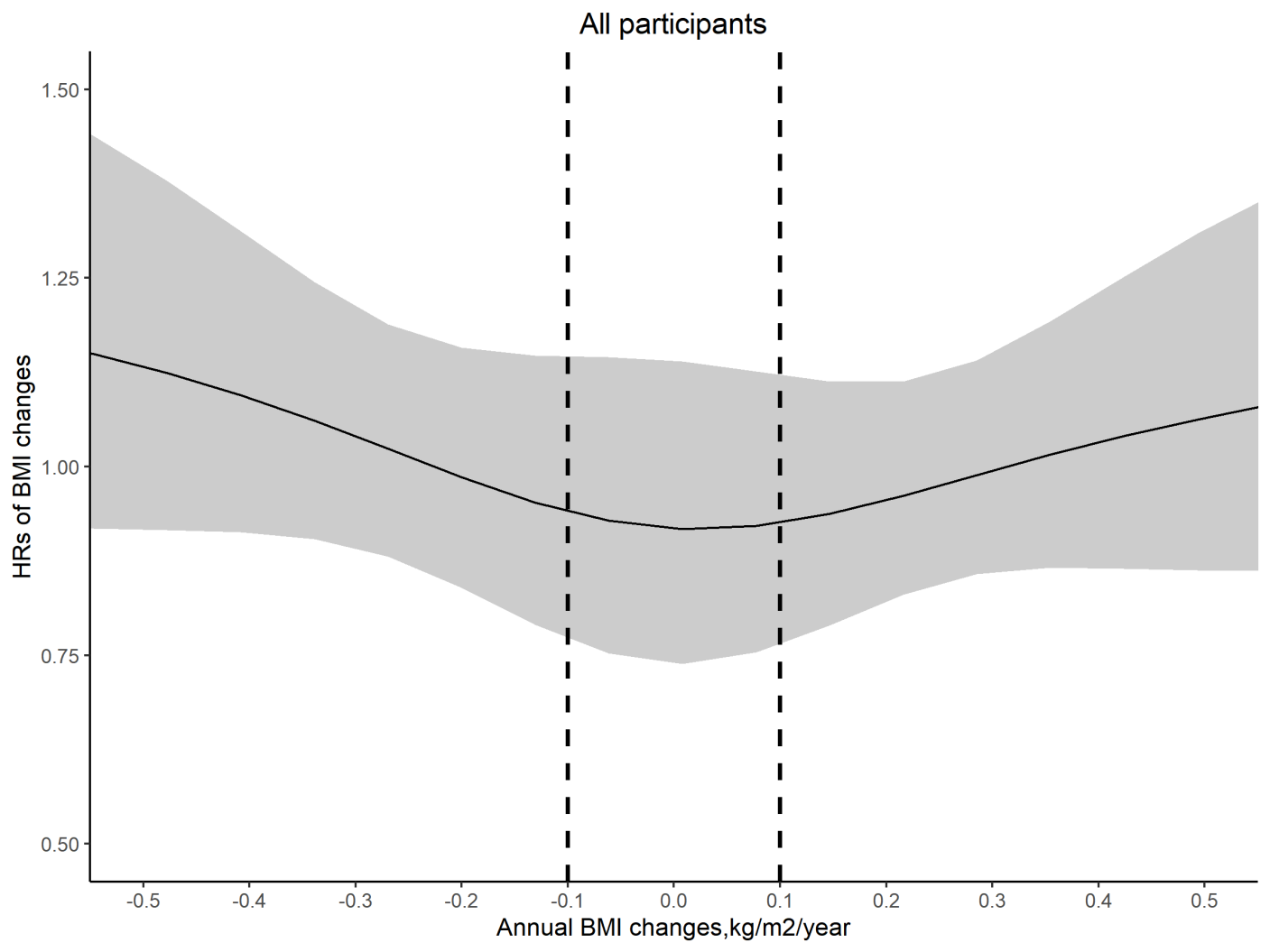


**Supplement Figure 2.** Restricted spline curves for the associations between annual change in BMI(continuous) and lung cancer incidence among all participants.
